# “Core/Shell” Nanocomposites as Photocatalysts for the Degradation of the Water Pollutants Malachite Green and Rhodamine B

**DOI:** 10.3390/ijms25126755

**Published:** 2024-06-19

**Authors:** Joana Zaharieva, Martin Tsvetkov, Milena Georgieva, Dimitar Tzankov, Maria Milanova

**Affiliations:** 1 Faculty of Chemistry and Pharmacy, Sofia University “St. Kliment Ohridski”, 1, J. Bourchier, 1164 Sofia, Bulgaria; nhjz@chem.uni-sofia.bg; 2 Faculty of Physics, Sofia University “St. Kliment Ohridski”, 5a, J. Bourchier, 1164 Sofia, Bulgaria; mgeorgieva@phys.uni-sofia.bg (M.G.); dtzankov@phys.uni-sofia.bg (D.T.)

**Keywords:** ferrites, nanocomposites, photocatalysis, pollutants degradation, magnetic properties

## Abstract

“Core/shell” composites are based on a ferrite core coated by two layers with different properties, one of them is an isolator, SiO_2_, and the other is a semiconductor, TiO_2_. These composites are attracting interest because of their structure, photocatalytic activity, and magnetic properties. Nanocomposites of the “core/shell” МFe_2_O_4_/SiO_2_/TiO_2_ (М = Zn(II), Co(II)) type are synthesized with a core of MFe_2_O_4_ produced by two different methods, namely the sol-gel method (SG) using propylene oxide as a gelling agent and the hydrothermal method (HT). SiO_2_ and TiO_2_ layer coating is performed by means of tetraethylorthosilicate, TEOS, Ti(IV) tetrabutoxide, and Ti(OBu)_4_, respectively. A combination of different experimental techniques is required to prove the structure and phase composition, such as XRD, UV-Vis, TEM with EDS, photoluminescence, and XPS. By Rietveld analysis of the XRD data unit cell parameters, the crystallite size and weight fraction of the polymorphs anatase and rutile of the shell TiO_2_ and of the ferrite core are determined. The magnetic properties of the samples, and their activity for the photodegradation of the synthetic industrial dyes Malachite Green and Rhodamine B are measured in model water solutions under UV light irradiation and simulated solar irradiation. The influence of the water matrix on the photocatalytic activity is determined using artificial seawater in addition to ultrapure water. The rate constants of the photocatalytic process are obtained along with the reaction mechanism, established using radical scavengers where the role of the radicals is elucidated.

## 1. Introduction

In recent years, investigations on the purification of polluted waters have been associated with the materials applied and the processes for pollutant elimination. Research on water’s purification by photocatalysis started by testing mainly TiO_2_ as a photocatalyst under UV light irradiation [1], both pure [2] and modified [3], followed by other oxides such as ZnO, NiO, and CeO_2_ [4,5,6,7,8,9,10,11], as well as sulfides such as CdS, ZnS [12,13], and mixed-metal oxides with spinel structure of the type MFe_2_O_4_ [14,15,16]. Mixed-metal oxides of the Zn_1−x_Ni_x_Fe_2_O_4_ type with a spinel structure have been tested for Malachite Green (MG) decomposition [17], methylene blue (MB) dye degradation under visible light with degradation efficiency increasing with Ni(II) content [18], Ni_x_Co_1 − x_Fe_2_O_4_ for MB decomposition [19], and Co_x_Mg_1 − x_Fe_2_O_4_ for photocatalytic degradation of MB both under sunlight and simulating visible light, where it was found that photocatalytic activity can be limited by an excessive amount of Co(II) [20], etc. Investigations on mixed-metal oxides as photocatalysts have developed in the direction of their incorporation into composites. Composites possessing so-called “core–shell” structures contain a mixed-metal oxide, very often ferrite, as a core in order to ensure magnetic functionality. In order to vary the functionality, quite often, the shell is made of different materials, among them ferrites, perovskites, silica, titania, noble metals, polymers, etc. [21]. For example, M_0.5_Fe_2.5_O_4_ (M = Co, Mn, and Ni) and Fe_3_O_4_ have been experimented with, where both ferrite and magnetite have been tested as the core and shell [22]. Similar core/shell structures CoFe_2_O_4_/ZnFe_2_O_4_ and, vice versa, ZnFe_2_O_4_/CoFe_2_O_4_, have been synthesized for tunable magnetic properties [23]. The variations of core/shell combinations, as well as the methods for their synthesis, their properties, and their area of application, including photocatalytic activity, are summarized in [21]. Among all of the compositions reviewed [21], only a few of them, containing a core coated with two consecutive layers/shells made of compounds differing in properties, have been studied, such as CoFe_2_O_4_/SiO_2_/TiO_2_ [24,25,26], CoFe_2_O_4_/SiO_2_/Y_2_O_3_:Tb^3+^ [27], and MnFe_2_O_4_/SiO_2_/CeO_2_ [28]. Along with the investigation of luminescence [27] and magnetic properties [24,25,26], including photocatalytic activity for the degradation of dyes such as dichlorophenol–indophenol [25], MB [24,26,28] has been studied under UV light irradiation. It has been shown that “core–shell” structured materials could improve photocatalytic activity in different ways, such as enhancing surface reactions, efficient charge transfer at the heterojunction interface, band structure modifications, and by improving the surface area [29].

The introduction of a SiO_2_ layer between the ferrite core of the MFe_2_O_4_ type and the TiO_2_ layer aims to reduce the recombination process between the TiO_2_ layer and the ferrite, as SiO_2_ could act as an insulator [24,30]. SiO_2_ is used as a middle layer due to its abundant –OH on the surface, as well as its high chemical and mechanical stability [27].

One of the challenges with “core/shells” of the MFe_2_O_4_/SiO_2_/TiO_2_ type is to evidence the phase composition and the structure, especially to prove the presence of TiO_2_ on the surface, taking into account its crucial role in the photocatalytic process. A suitable combination of experimental techniques is needed, such as TEM/EDS and XPS. Another issue to be mentioned is related to the polymorphs of TiO_2_, anatase and rutile, especially their ratio. The latter can be observed by Rietveld analysis based on XRD data. Actually, the Rietveld analysis of XRD data is not a very commonly applied approach, especially not in the MFe_2_O_4_/SiO_2_/TiO_2_ characterization discussed so far. Anatase, rutile, and brookite are the crystalline phases of TiO_2_. Of those, anatase has shown the highest photocatalytic activity [31,32], usually produced by the calcination of TiO_2_ at around 500–600 °C [33]. At that temperature, mixed-metal oxides, such as cobalt ferrite and zinc ferrite, are known to be thermally stable compounds.

**Figure 1:**
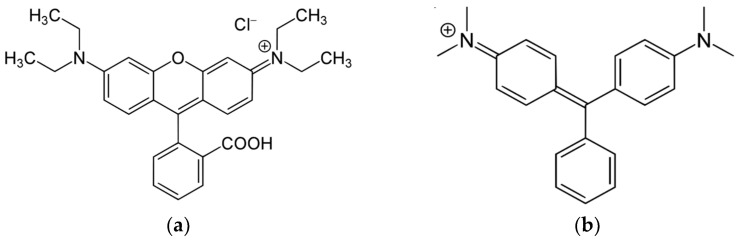
The structures of (**a**) Rhodamine B and (**b**) Malachite Green.

The purpose of the work presented is to obtain nanocomposites of the “core/shell” МFe_2_O_4_/SiO_2_/TiO_2_ (M = Zn(II), Co(II)) type, where a layer of titania is deposited on nanoparticles of metal ferrite with pre-deposited silica in order to study their structure and phase composition by different methods, including Rietveld analysis of XRD data, as well as to study their properties, both photocatalytic activity under UV-light and simulated solar irradiation and magnetic properties. The latter is to be expected due to the МFe_2_O_4_ ferrites used as a core. Two organic dyes different from those used in [24,25,26] are selected as model pollutants in water solutions. Rhodamine B, RhB (Figure 1a), is widely used in the textile industry and is a potential cause for carcinogenic and teratogenic effects, but is known for its good stability [2,34]. The other one is the Malachite Green, MG (Figure 1b), a basic, synthetic organic dye, soluble in water with antimicrobial properties, used for the dyeing of leather, wool, silk, paper, etc., as well as for the control of fungal and bacterial infections of fish, having mutagenic and carcinogenic properties [35].

The selection of the dyes tested in this study was based on (i) their easy detection by UV/Vis spectrophotometry, at 550 nm for Rh B and 622 nm for MG, respectively; (ii) the convenience of the preparation of the model solutions for photocatalytic tests; (iii) previous experience of their decomposition using other photocatalysts synthesized by the authors [3,17].

The influence of the water matrix on the photocatalytic activity is followed by the testing of dye solutions both in ultrapure and artificial seawater. The recycling and stability of the composite prepared after recycling is investigated.

## 2. Results

### 2.1. Core/Shell Structured Composites Based on Sol-Gel (SG)-Produced CoFe_2_O_4_

#### 2.1.1. Phase Composition and the Crystal Structure of CoFe_2_O_4_, Synthesized by the Sol-Gel Method (SG), and of the Composite CoFe_2_O_4_/SiO_2_/TiO_2_ (SG) Based on It

**Figure 2:**
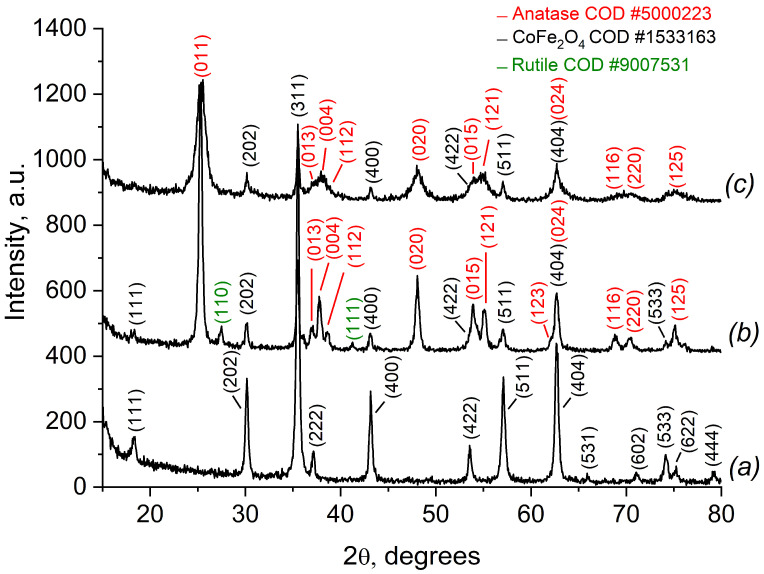
XRD patterns of (**a**) CoFe_2_O_4_ (SG), (**b**) CoFe_2_O_4_/TiO_2_ (SG), and (**c**) CoFe_2_O_4_/SiO_2_/TiO_2_ (SG).

According to the XRD pattern (Figure 2a), the CoFe_2_O_4_ prepared by the sol-gel method has a well-crystallized single phase of the face-centered cubic spinel structure (space group Fd3m).

The calculated unit cell parameter (Table 1) is in good agreement with crystallographic databases (8.3550 Å, COD #00-591-0063).

The XRD pattern of the nanocomposite CoFe_2_O_4_/TiO_2_ (SG) (Figure 2b) shows pure CoFe_2_O_4_ with diffraction peaks of TiO_2_, anatase, and rutile. The unit cell parameters and the crystallite size were calculated using Rietveld’s method. The unit cell calculation shows that, for anatase, a = 3.7833 and c = 9.4391 Å, for rutile, a = 4.5815 and c = 2.9531 Å, and for CoFe_2_O_4_, a = 8.3652 Å. The XRD pattern of the nanocomposite CoFe_2_O_4_/SiO_2_/TiO_2_ (SG) displays the presence of anatase only (Figure 2c) with unit cell parameters for anatase a = 3.7893 and c = 9.4364 Å, and for CoFe_2_O_4_, a = 8.3631 Å. A slight tendency toward increasing the crystallite size is observed for CoFe_2_O_4_, i.e., the pure one has a crystallite size of 48 nm, while the one included in the nanocomposites has a size of 56 and 53 nm, respectively, for CoFe_2_O_4_/TiO_2_ (SG) and CoFe_2_O_4_/SiO_2_/TiO_2_ (SG). The potential reason for this tendency is the secondary crystal growth caused by additional thermal treatment during TiO_2_ formation. A tendency toward decreasing the crystallite size is observed for TiO_2_ (anatase): 25 nm for CoFe_2_O_4_/TiO_2_ (SG) and 16 nm for CoFe_2_O_4_/SiO_2_/TiO_2_ (SG), respectively.

The nanocomposites CoFe_2_O_4_/TiO_2_ (SG) and CoFe_2_O_4_/SiO_2_/TiO_2_ (SG) show different phase compositions of the TiO_2_ layer, i.e., CoFe_2_O_4_/TiO_2_ (SG) contains both polymorphs, anatase and rutile, but for CoFe_2_O_4_/SiO_2_/TiO_2_ (SG), only anatase is detected (Table 1). It is known that different experimental conditions influence the transformation of the metastable anatase phase of TiO_2_ to the equilibrium rutile phase, among which are the synthetic procedure, the initial material, the calcination time, the atmosphere of calcination, some additives, etc. [36]. Upon heating, the A/R (anatase/rutile) ratio depends on the temperature applied; for example, rutile contents (%) of 0 (500 °C), 46 (600 °C), and 94 (700 °C) are reached for sol-gel-produced TiO_2_ [37]. In our case, in addition to the annealing temperature, the presence of SiO_2_ also influences the transformation A/R ratio. It can be assumed that the phase transition of anatase to rutile is limited by the presence of SiO_2_. As a result, at 550 °C, only anatase was observed, while in the samples without SiO_2_ layer, TiO_2_ polymorph anatase partially transformed into rutile.

#### 2.1.2. Morphology of the Composites CoFe_2_O_4_/SiO_2_/TiO_2_ (SG)

**Figure 3:**
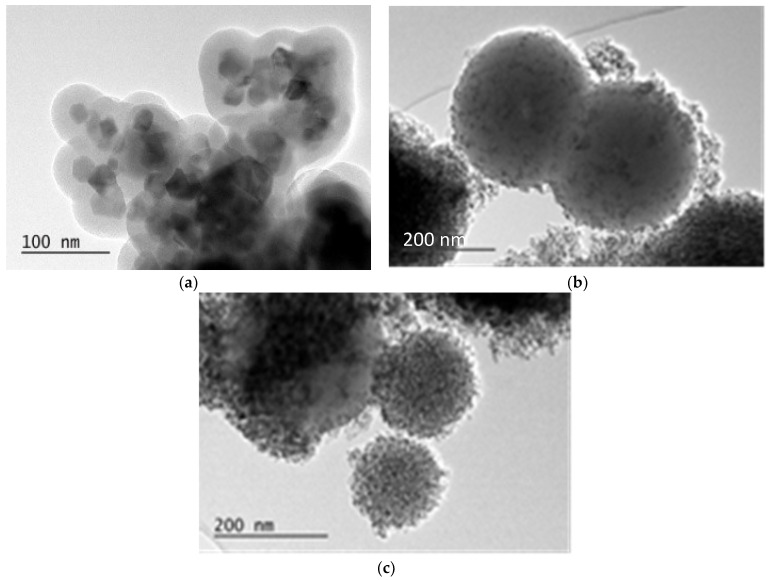
ТЕМ micrographs of core/shell composites: (**a**) CoFe_2_O_4_/SiO_2_ (SG) and (**b,c**) CoFe_2_O_4_/SiO_2_/TiO_2_ (SG).

The morphology and the structure of as-synthesized composites can be observed in the transmission electron microscopy graphs (TEM) (Figure 3). This is significant, particularly for the one-layered core–shell CoFe_2_O_4_/SiO_2_ (SG), where XRD is not informative because it displays an amorphous sample. According to the information obtained by TEM, CoFe_2_O_4_/SiO_2_ (SG) contains a very well-crystallized core of CoFe_2_O_4_ with a well-shaped covering layer of SiO_2_ (Figure 3a and Figure S1). In spite of some aggregation observed, the structure of a single core covered with a shell can be detected. Regarding the thickness of the SiO_2_ layer, it can be estimated to be, on average, approximately 25–30 nm.

After the CoFe_2_O_4_/SiO_2_ composite is coated with TiO_2_ by the procedure presented in Section 4.2.3, the CoFe_2_O_4_/SiO_2_/TiO_2_ (SG) composite is formed. The latter can be seen in the TEM micrographs (Figure 3b,c), where TiO_2_ coating on the surface of silica spheres can be observed. The size of the CoFe_2_O_4_/SiO_2_ spheres (Figure 3a) is approximately 100 nm in diameter, while the size of the CoFe_2_O_4_/SiO_2_/TiO_2_ (SG) spheres (Figure 3b,c) is bigger, roughly 150–300 nm. That difference in the size could be explained by the presence of the TiO_2_ layer on CoFe_2_O_4_/SiO_2_/TiO_2_. In Figure 3a, the CoFe_2_O_4_ core is very well formed and visible, and the cobalt ferrite core of CoFe_2_O_4_/SiO_2_/TiO_2_ (SG) (Figure 3b,c) is not visible. Quite likely, the reason is that the dense layer of TiO_2_ disturbs the electron beam penetration.

**Figure 4:**
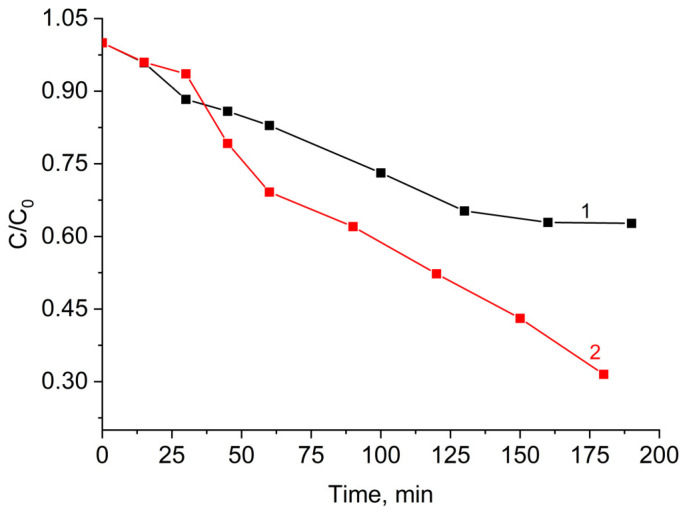
Photocatalytic degradation of Malachite Green by the nanocomposites (black, 1) CoFe_2_O_4_/TiO_2_ (SG) and (red, 2) CoFe_2_O_4_/SiO_2_/TiO_2_ (SG) under UV irradiation.

The experiments proved that, under UV illumination, the two-layered core–shell composite CoFe_2_O_4_/SiO_2_/TiO_2_ (SG) has better photocatalytic activity for MG decomposition with a rate constant of 5.8 × 10^−3^ min^−1^, in comparison with the activity of one-layered CoFe_2_O_4_/TiO_2_ (SG) with a rate constant 2.9 × 10^−3^ min^−1^ (Figure 4). It should be mentioned that the activity detected is lower than the activity of TiO_2_ P25 under the same experimental conditions, with a rate constant of 12.0 × 10^−3^ min^−1^ obtained, for instance, in [38].

### 2.2. “Core/Shell” Structured Composites MFe_2_O_4_/SiO_2_/TiO_2_ (M = Zn, Co) Based on Hydrothermally (HT) Produced CoFe_2_O_4_ and ZnFe_2_O_4_

#### 2.2.1. Phase Composition of MFe_2_O_4_/SiO_2_/TiO_2_ (M = Zn, Co), with CoFe_2_O_4_ and ZnFe_2_O_4_ Produced via the Hydrothermal (HT) Method

**Figure 5:**
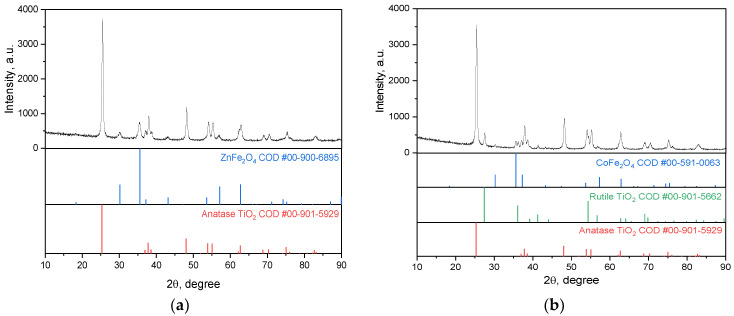
The comparison of XRD data for the synthesized composites (**a**) ZnFe_2_O_4_/SiO_2_/TiO_2_ (HT) and (**b**) CoFe_2_O_4_/SiO_2_/TiO_2_ (HT) with the referents of the COD data for MFe_2_O_4_ (M = Zn, Co) (top), rutile (middle), and anatase (bottom).

According to the XRD pattern (Figure 5a,b, the top), both CoFe_2_O_4_ and ZnFe_2_O_4_ prepared by the HT method have well-crystallized phases. The unit cell parameters calculated (Table 2) are in good agreement with crystallographic databases (COD # 00-900-6895 and 00-591-0063, for ZnFe_2_O_4_ and CoFe_2_O_4_, respectively). No change in the unit cell parameter for MFe_2_O_4_ after adding the layers (Table 2) can be expected. The XRD pattern of the ZnFe_2_O_4_/SiO_2_/TiO_2_ (HT) nanocomposite only shows the presence of anatase (Figure 5a, the bottom) with unit cell parameters after calculation for anatase, a = b = 3.7899 and c = 9.5230 Å, and for ZnFe_2_O_4_, a = b = c = 8.4463 (9) Å. For CoFe_2_O_4_/SiO_2_/TiO_2_ (HT), both rutile and anatase are registered (Figure 5b, middle and bottom, correspondingly). The unit cell parameter calculations for CoFe_2_O_4_ are a = b = c = 8.3874 Å, for rutile, a = b = 4.5953 (6), c = 2.9608 (7) Å, and for anatase, a = b = 3.7860 and c = 9.5185 Å. For MFe_2_O_4_ (M = Zn, Co), included in the nanocomposites, the crystallite size is 19 and 34 nm, for ZnFe_2_O_4_/SiO_2_/TiO_2_(HT) and CoFe_2_O_4_/SiO_2_/TiO_2_ (HT), respectively (Table 2).

**Figure 6:**
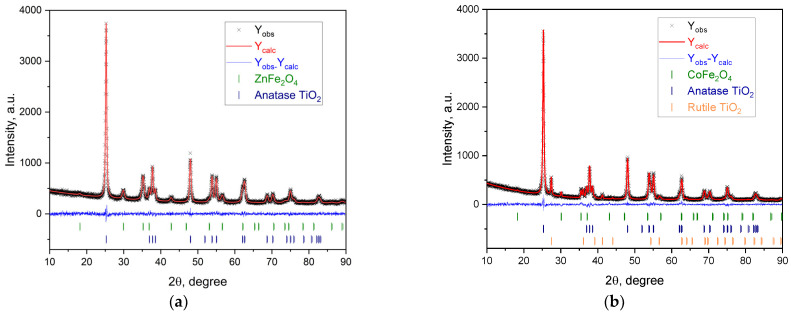
Experimentally observed (black dots), Rietveld calculated (continuous red line), and difference (continuous bottom blue line) profiles obtained after Rietveld analysis of the XRD data of (**a**) ZnFe_2_O_4_/SiO_2_/TiO_2_ (HT) (M = Zn, Co) and (**b**) CoFe_2_O_4_/SiO_2_/TiO_2_ (HT). Peak positions are shown at the baseline as small markers.

The Rietveld analysis of the XRD data of the composites MFe_2_O_4_/SiO_2_/TiO_2_ (M = Zn, Co) was used to gain additional information about the structural and microstructural characteristics of the samples. The crystalline phase ratios were also observed (Figure 6).

In Table 2, the weight fraction (%) of the components of the composites is shown. The synthetic procedure followed for the ferrite MFe_2_O_4_ (M = Zn, Co) is the same, but the weight of CoFe_2_O_4_ detected is low. Due to the magnetic field caused by the magnetic stirrer used (Section 4.2.2), during the coating of CoFe_2_O_4_ with SiO_2_, some of the CoFe_2_O_4_ sticks to the stirrer. CoFe_2_O_4_ can be easily separated by the composite sample formed. Its amount is reproducible due to the same experimental conditions used, i.e., the initial amount of CoFe_2_O_4_, the power of the magnetic stirrer, and the size of the magnetic anchor.

**Figure 7:**
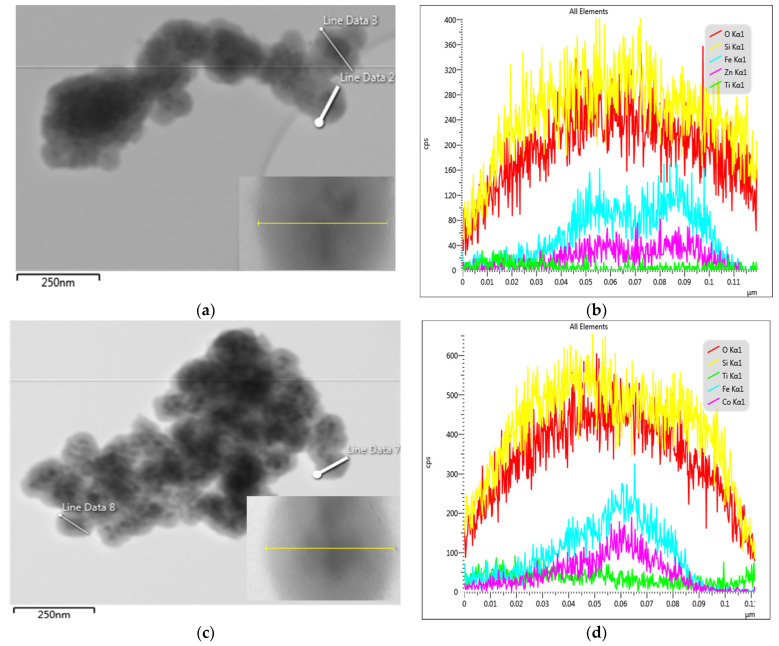
TEM/EDS of (**a**,**b**) ZnFe_2_O_4_/SiO_2_/TiO_2_ and (**c**,**d**) CoFe_2_O_4_/SiO_2_/TiO_2_. The line scan is presented by the yellow line in the inset.

**Figure 8:**
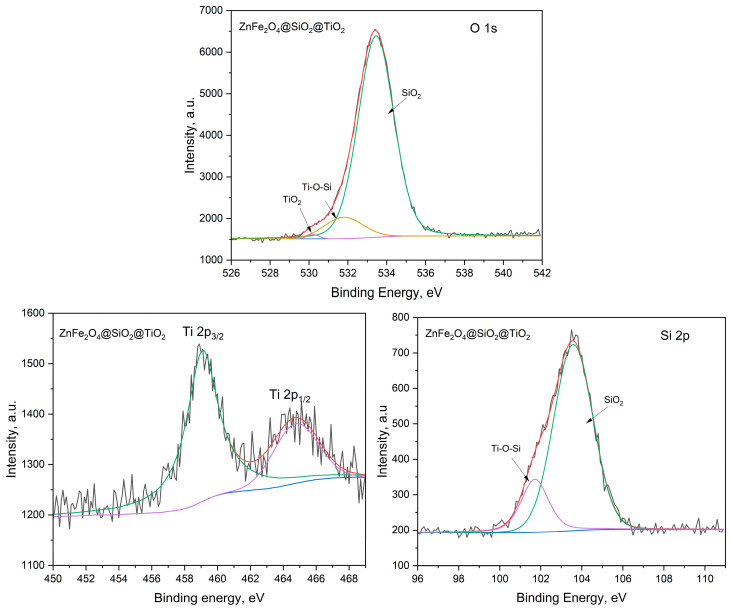
ZnFe_2_O_4_/TiO_2_/SiO_2_ (HT): high-resolution XPS spectra of O1s, Ti2p, and Si2p.

**Figure 9:**
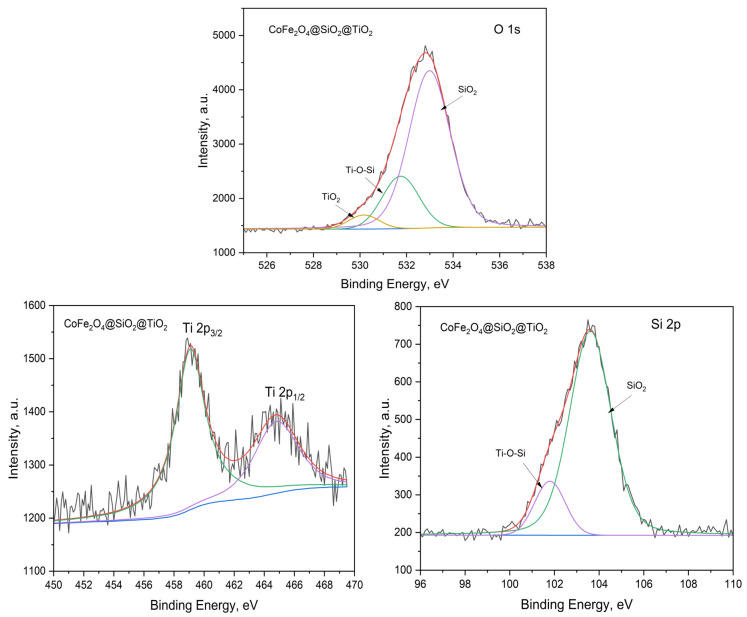
CoFe_2_O_4_/TiO_2_/SiO_2_ (HT): high-resolution XPS spectra of O1s, Ti2p, and Si2p.

The morphology of the ZnFe_2_O_4_/SiO_2_/TiO_2_ (HT) and CoFe_2_O_4_/SiO_2_/TiO_2_ (HT) composites characterized by TEM/EDS is presented in Figure 7. The agglomerates and well-shaped spheres can be seen (Figure 7a,c). In order to prove the core–shell structure and phase composition, a line scan through a well-defined particle was performed (shown in the insets). By that, it was established that the darker area (the core) is the well-crystalized ferrite (consisting of Fe, Zn/Co) surrounded by a relatively thick layer of SiO_2_ and a very thin uniform layer of TiO_2_ (Figure 7b,d). The results are consistent with the conclusions from the XPS analysis, confirming the presence of the thin TiO_2_ shell on the top of the thicker SiO_2_ layer (Figure 8 and Figure 9).

To prove the elemental composition of “core/shell” structures, X-ray photoelectron spectroscopy (XPS) was applied. The characteristic peaks of the surface atoms in the XPS spectra were registered, and based on the energy of these peaks, the identification of the surface elements was carried out (Figure S2). All core-level spectra were referenced to the C1s hydrocarbon peak at 284.5 eV to compensate for surface charging (internal charge correction [39]). Based on the XPS spectrum of the ZnFe_2_O_4_/SiO_2_/TiO_2_ (HT) composite, the following comments can be added (Figure 8): due to its asymmetry, the O1s peak can be deconvoluted to three components (three different oxygen species). The peaks at 533.4, 531.3, and 530.2 eV can be ascribed to Si-O-Si in SiO_2_ and Ti-O-Si and Ti-O-Ti in TiO_2_ [40,41]. This indicates (i) a strong interaction between the SiO_2_ and TiO_2_ layers and (ii) an extremely thin TiO_2_ layer (taking into account the integral area of this peak compared to the rest), which is in a good correlation with the results from the TEM/EDS analysis. The location of Ti2p3/2 at 459.1 eV and the splitting of 5.7 eV between Ti2p3/2 and Ti2p1/2, indicate the Ti(IV) oxidation state [42]. The peaks at 103.6 and 101.7 eV can be attributed to the Si-O bond in SiO_2_ and Si-O-Ti, respectively [43].

The XPS spectrum of CoFe_2_O_4_/SiO_2_/TiO_2_ closely resembles that of the ZnFe_2_O_4_/SiO_2_/TiO_2_ composite (Figure 9). The O1s peaks are located at 533.0, 531.7, and 530.2 eV for Si-O-Si in SiO_2_ and Ti-O-Si and Ti-O-Ti in TiO_2_, respectively. As expected, the Si2p peaks are located at 103.6 and 101.8 eV for the Si-O bond in SiO_2_ and Si-O-Ti, respectively. Accordingly, Ti2p3/2 is also located at 459.1 eV, while Ti2p1/2 is at 464.4 eV (peak separation of 5.3 eV), which indicates the Ti(IV) oxidation state. A difference in the XPS spectra of both composites was detected in the overall integral area of the peak corresponding to TiO_2_, which is higher in CoFe_2_O_4_/SiO_2_/TiO_2_ than in the ZnFe_2_O_4_ SiO_2_/TiO_2_ composite. This result correlates well with the XRD data.

The characteristic peaks for Zn, Co, and Fe atoms were not registered in the XPS spectra of the composites, i.e., those are the atoms of the core ferrites (Figure S2). XPS is a surface-sensitive technique. The X-ray penetration through SiO_2_/TiO_2_ layers is several µm, while the thickness of the SiO_2_ layer is approximately 25–30 nm (as commented in Section 2.1.2., page 5, for example). The attempts to register Zn, Co, and Fe atoms were unsuccessful. The reason is that the photoelectron escape depth is approximately 10 nm or less, so only those escaping the samples without energy loss can contribute to the XPS [39].

#### 2.2.2. Optical Properties and the Energy Band Gap of MFe_2_O_4_/SiO_2_/TiO_2_ with MFe_2_O_4_ (M = Zn, Co) when HT-Synthesized

**Figure 10:**
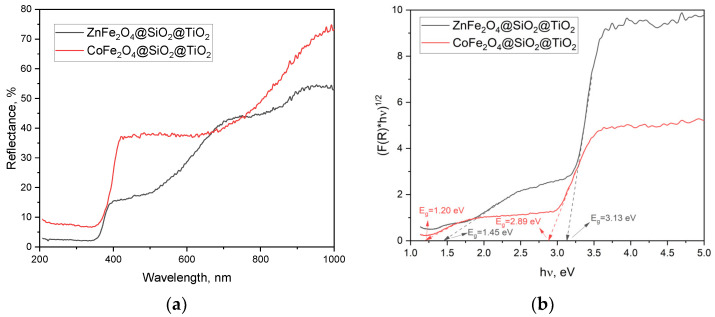
(**a**) Reflectance of the samples ZnFe_2_O_4_/SiO_2_/TiO_2_(HT) and CoFe_2_O_4_/SiO_2_/TiO_2_(HT); (**b**) energy band gap calculated on the base of the reflectance.

The reflectance of light by the powdered samples ZnFe_2_O_4_/SiO_2_/TiO_2_(HT) and CoFe_2_O_4_/SiO_2_/TiO_2_(HT) was registered in the region of 200–1000 nm (Figure 10a). Based on that, the energy band gap was calculated. Two values were obtained for ZnFe_2_O_4_/SiO_2_/TiO_2_(HT) (1.45 and 3.13 eV) and CoFe_2_O_4_/SiO_2_/TiO_2_(HT) (1.20 and 2.89 eV) (Figure 10b). The value of 1.20 eV obtained for CoFe_2_O_4_ is close to 1.22 eV, obtained by the sol-gel method, reported in [17]. The values of 3.13 and 2.89 eV are consistent with the band gap of anatase and the phase A/R ratio, respectively, knowing that the anatase band gap energy of 3.22 eV is broader than that for rutile, 3.0 eV. According to [43], the hydrothermal conditions could affect the band gap energy of TiO_2_ polymorphs.

#### 2.2.3. Photocatalytic Activity of the MFe_2_O_4_/SiO_2_/TiO_2_ (HT) with MFe_2_O_4_ (M = Zn, Co) Composites when HT-Synthesized

**Figure 11:**
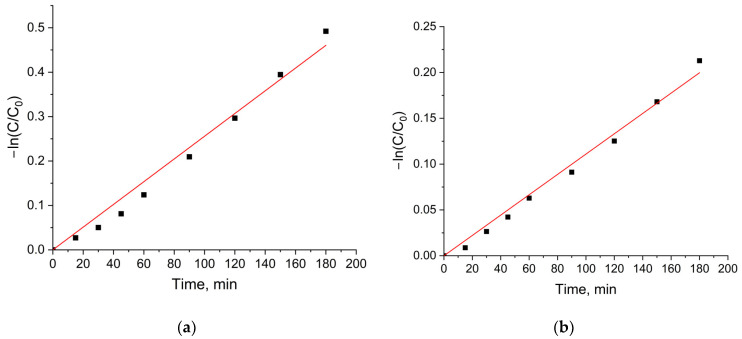
Kinetic curves of the decomposition of (**a**) MG and (**b**) RhB in the presence of the composite CoFe_2_O_4_/SiO_2_/TiO_2_ (HT) under UV irradiation.

Under UV light irradiation, the photocatalytic activity of CoFe_2_O_4_/SiO_2_/TiO_2_(HT) is lower for the degradation of both MG and RhB, taking into account the rate constants 2.6 × 10^−3^ min^−1^ and 1.11 × 10^−3^ min^−1^, correspondingly (Figure 11). The values of the rate constants are lower than those obtained with TiO_2_ P25 under the same experimental conditions, for instance, 12.0 × 10^−3^ min^−1^ in [38].

**Figure 12:**
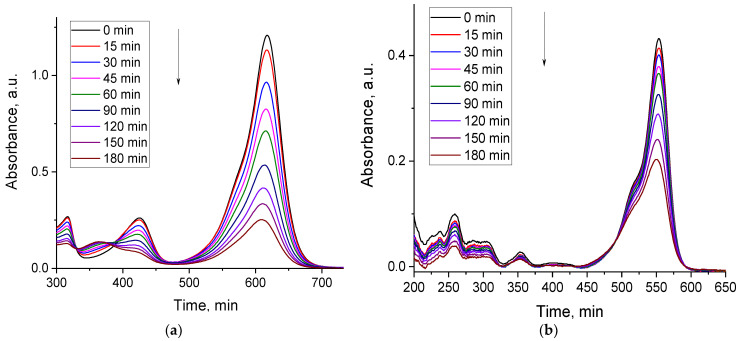
Photocatalytic degradation of the dyes (**a**) MG and (**b**) RhB under UV light irradiation in the presence of ZnFe_2_O_4_/SiO_2_/TiO_2_(HT) evaluated by the absorbance. The arrow points decreasing of the absorbance with the irradiation time.

Under UV light irradiation, the composite ZnFe_2_O_4_/SiO_2_/TiO_2_ (HT) shows activity against two dyes, MG and RhB, with degradation for MG at 80% and for RhB at 53%. The values are obtained by the dye’s absorbance, recorded by UV/Vis absorption spectroscopy (Figure 12).

The results of the degradation of the dyes determined by UV/Vis absorption (monitoring the band at 622 for MG and at 550 nm for RhB) are different compared to the results obtained by total organic carbon, TOC. Тhe decomposition of the organic molecules to CO_2_ and H_2_O proved by TOC is 39.7% for RhB, but no degradation of MG was registered. Using UV/Vis spectroscopy, the targeted pollutant is the only one to be detected, while the intermediate products (possibly more toxic than the original molecules themself) cannot be detected. At the same time, by TOC, the complete mineralization of an organic molecule can be followed. The TOC measurements show lower values for MG removal than UV/Vis spectroscopy. This is a clear indication that mineralization goes through a complex multistep process, including several intermediate products. For example, 16 intermediate products have been identified during the photocatalytic oxidation of MG over α-NiMoO_4_ [44]. All byproducts have different oxidation potentials when reacting with the radicals produced during the photocatalytic process. This leads to different speeds of mineralization for each of the intermediates and, therefore, their detection during TOC analysis.

If the ZnFe_2_O_4_/SiO_2_/TiO_2_ (HT) composite was more active under UV light, its activity was tested under simulated solar irradiation in a seawater matrix and for recycling.

**Figure 13:**
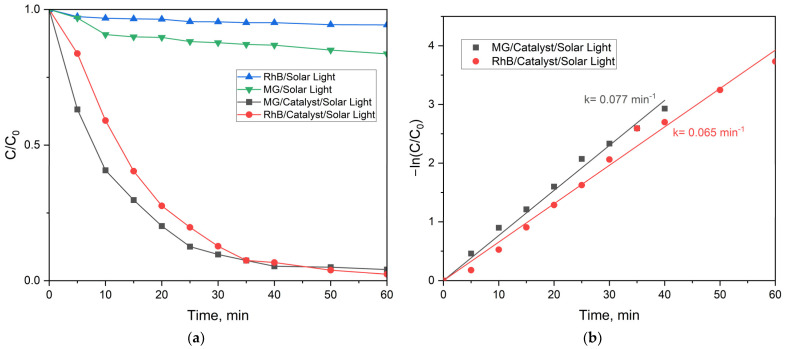
(**a**) Kinetic curves of the photolysis of RhB (blue) and MG (green) and the photocatalytic decomposition of MG (black) and RhB (red) in the presence of ZnFe_2_O_4_/SiO_2_/TiO_2_ (HT) under simulated solar irradiation; (**b**) rate constants of the process of RhB and MG degradation under simulated solar irradiation.

MG and RhB dyes are stable under simulated solar irradiation. As can be seen, no photolysis was observed (Figure 13a). At the same time, the dyes were almost completely decomposed in the presence of the ZnFe_2_O_4_/SiO_2_/TiO_2_ (HT) composite under simulated solar light for 60 min irradiation. An efficiency of degradation of 98% was achieved, indicating the significantly higher photocatalytic activity of ZnFe_2_O_4_/SiO_2_/TiO_2_ (HT) under solar irradiation than under UV light irradiation. The rate constant obtained for the degradation of MG dye was higher than for RhB, 0.077 min^−1^ and 0.065 min^−1^, respectively, for MG and RhB (Figure 13b). Additionally, the TOC data showed very high mineralization efficiency, 92% for MG and 88% for RhB. Overall, the activity of the ZnFe_2_O_4_/SiO_2_/TiO_2_(HT) composite is significantly higher under simulated solar irradiation (300 W lamp) compared to UV-light irradiation alone (18 W lamp). It should be taken into account that the solar simulator used has a spectral distribution that is stronger than that of the sun in the UV region of the spectrum [45]. Spectral radiation distribution shows a high portion of the UV radiation. Its radiated power in the 315–400 nm (UVA) region is 13.6 W, and in the 280–315 nm (UVB) region, it is 3.0 W [46].

The influence of the water matrix on the photocatalytic activity of ZnFe_2_O_4_/SiO_2_/TiO_2_ (HT) was investigated in artificial seawater both under UV light and under simulated solar light.

**Figure 14:**
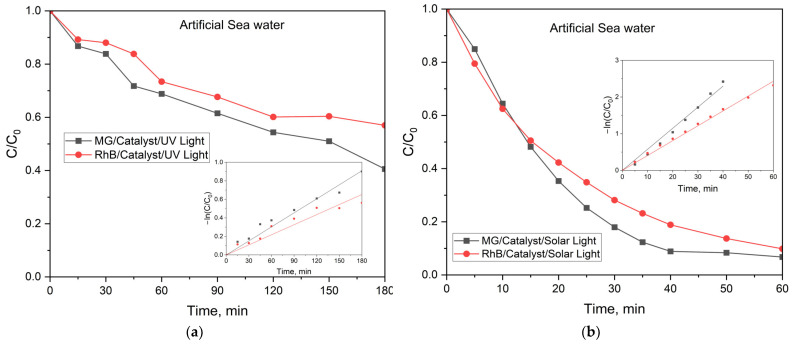
Photocatalytic activity of ZnFe_2_O_4_/SiO_2_/TiO_2_ (HT) in artificial seawater: (**a**) under UV light and (**b**) under simulated solar irradiation for MG (black) and RhB (red) (see the legend).

The results from the photocatalytical experiments under UV light in seawater are presented in Figure 14a. The photocatalytic activity of the ZnFe_2_O_4_/SiO_2_/TiO_2_ (HT) composite is lower in seawater than in ultrapure water. The maximum degradation efficiency at 180 min irradiation reached for MG dye is 59.5% (rate constant 5.1 × 10^−3^ min^−1^), while for RhB, it is 42% (rate constant 3.6 × 10^−3^ min^−1^).

Taking into account the high catalytic activity observed under simulated solar irradiation in ultrapure water (Figure 13), experiments in artificial seawater under solar light were also conducted, and the results are presented in Figure 14b. As can be seen, the ZnFe_2_O_4_/SiO_2_/TiO_2_ (HT) composite shows excellent photocatalytic activity under solar radiation, even in seawater. The degradation efficiency is slightly lower than that found in ultrapure water (93% degradation for MG and 90% for RhB) with lower rate constants (rate constant for MG is 5.7 × 10^−3^ min^−1^, while for RhB is 4.0 × 10^−3^ min^−1^). Despite that, the results show that the prepared ZnFe_2_O_4_/SiO_2_/TiO_2_ (HT) composite can be used in real water matrices (seawater) with less than 10% loss of activity for azo dyes.

The data showing the influence of the water matrix and the irradiation light on the photocatalytic activity of ZnFe_2_O_4_/SiO_2_/TiO_2_ (HT) are summarized in Table 3. The artificial seawater matrix inhibits the role of both the UV and simulated solar light. The ions present in the artificial seawater, such as chloride and sulfate, display a different influence on the oxidation process of the dye, probably reacting with the radicals responsible for the oxidation of the dyes [47].

**Figure 15:**
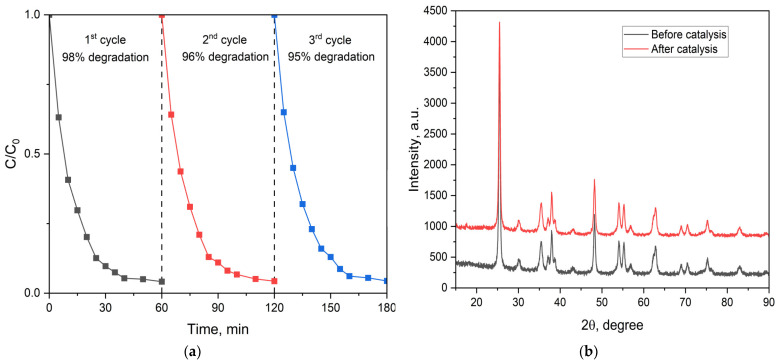
(**a**) Photocatalytic degradation of MG under solar irradiation with recycled ZnFe_2_O_4_/SiO_2_/TiO_2_ (HT); (**b**) XRD pattern of the ZnFe_2_O_4_/SiO_2_/TiO_2_ (HT) photocatalyst before and after photocatalytic experiments (solar irradiation, MG degradation).

Catalyst recycling experiments were performed using MG dye in ultrapure water and under solar irradiation due to the high photocatalytic activity of the prepared materials under these conditions. The ZnFe_2_O_4_/SiO_2_/TiO_2_ (HT) composite retained its high photocatalytic activity under solar irradiation for at least three consecutive cycles. The degradation efficiency diminished from 98 to 96 and 95% (Figure 15a). Furthermore, the XRD patterns of the photocatalyst before and after the photocatalytic experiments under solar irradiation are presented in Figure 15b, where no significant difference is observed between the as-prepared and spent photocatalyst, indicating that the phase composition remains the same.

#### 2.2.4. Mechanism of the Photocatalytic Process

**Figure 16:**
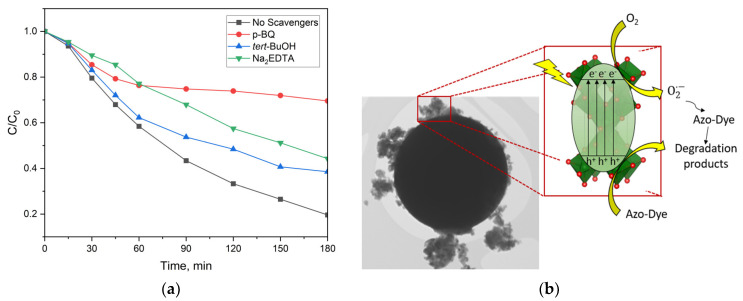
(**a**) Efficiency of the different radical scavengers: p-benzoquinone, tert-BuOH, and Na_2_EDTA for the degradation of azo dye (according to the legend). (**b**) Schematic illustration of the radicals and h^+^ holes formation and the degradation of azo dye (MG and RhB) under UV irradiation in the presence of CoFe_2_O_4_/SiO_2_/TiO_2_.

In order to establish the reaction mechanism of the photocatalytic process, a series of experiments were conducted using radical scavengers with a concentration fixed at 10 ppm. The scavengers p-benzoquinone for superoxide anion radicals O_2_^•−^, tert-butanol for OH- radicals [48], and Na_2_EDTA as a scavenger for surface-generated holes h^+^ [49] were tested. The results show that the major reactive species are the h^+^ holes and superoxide anion radicals O_2_^•−^ (Figure 16a). Based on the results, the reaction mechanism can be commented on. Briefly, by excitation, the surface of MFe_2_O_4_/SiO_2_/TiO_2_ (M = Co, Zn) with UV light electron-hole pairs is produced. The photogenerated electrons, e^−^, are transferred to the conduction band from the valence band, creating positive holes, h^+^. The photogenerated holes can directly degrade the azo dye (MG or RhB). At the same time, the photogenerated electrons can react with O_2_ to produce anion radicals O_2_^•−^, which, on their side, can degrade the azo dye (MG or RhB), as illustrated in Figure 16b.

**Figure 17:**
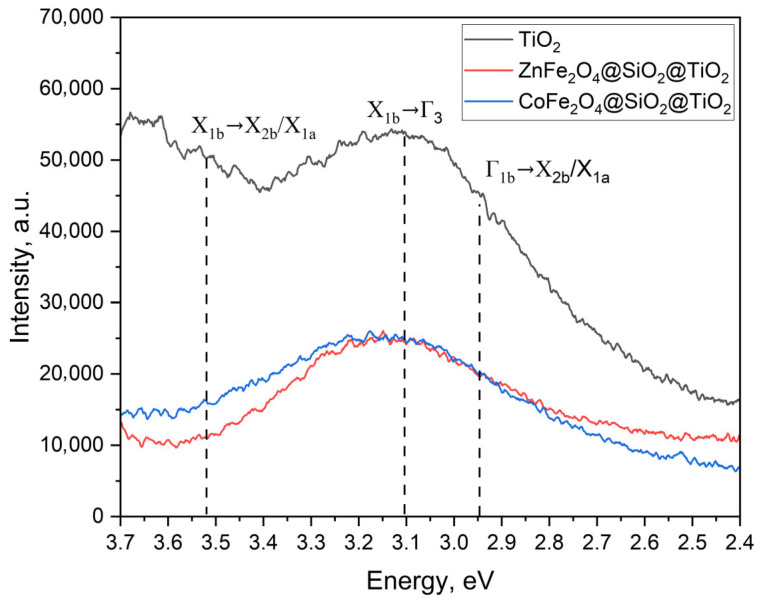
The photoluminescent spectra of pure TiO_2_ and MFe_2_O_4_/SiO_2_/TiO_2_ (M = Zn, Co).

In order to show the charge separation, the photoluminescent spectra of the obtained pure TiO_2_ (prepared by the procedure in Section 4.2.3.) and MFe_2_O_4_/SiO_2_/TiO_2_ (M = Zn, Co) were recorded (Figure 17) at 315 nm [50]. It can be seen that the emission spectrum of TiO_2_ can be deconvoluted into three main emission bands known to be typical for anatase TiO_2_ [51] and the emission band at ~3.50–3.53 eV can be assigned following the degenerated direct transition X_1b_ → X_2b_/X_1a_ (Figure 17). This transition is slightly visible in pure TiO_2_ and completely missing in the MFe_2_O_4_/SiO_2_/TiO_2_ composites (Figure 17). The most intense and broad band, due to the indirect transition X_1b_ → Γ_3_, is located at around 3.1 eV for pure TiO_2_ but is slightly red-shifted for composites. The lowest energy band between 2.9–3.0 eV (presented as a shoulder in all samples) is the nearly degenerated indirect transition Γ_1b_ → X_2b_/X_1a_. Overall, the intensity of all bands significantly decreases for the composites in comparison to TiO_2_, which is an indication of improved charge separation.

#### 2.2.5. Magnetic Measurements

**Figure 18:**
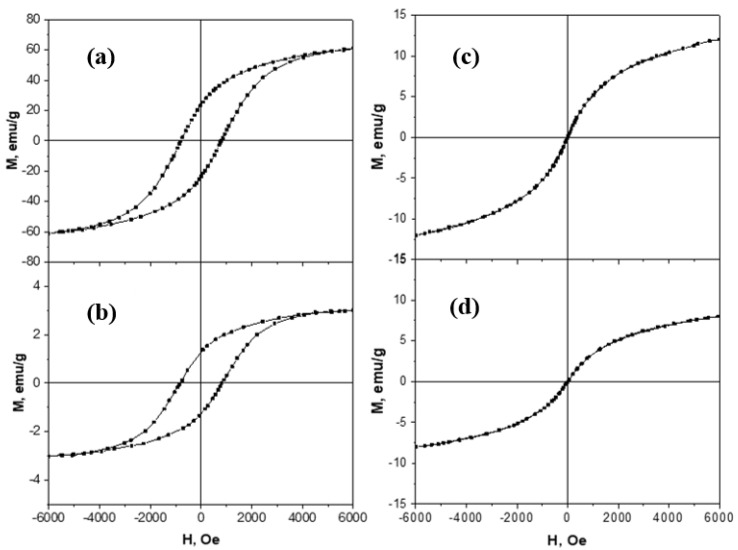
Magnetization versus magnetic field curves for the samples (**a**) CoFe_2_O_4_, (**b**) CoFe_2_O_4_/SiO_2_/TiO_2_(HT), (**c**) ZnFe_2_O_4_, and (**d**) ZnFe_2_O_4_/SiO_2_/TiO_2_(HT) measured by VSM at room temperature.

The magnetic properties of CoFe_2_O_4_ and the CoFe_2_O_4_/SiO_2_/TiO_2_ (HT) composite, as well as of ZnFe_2_O_4_ and the ZnFe_2_O_4_/SiO_2_/TiO_2_(HT) composite, were studied by a vibrating sample magnetometer (VSM) at room temperature. The cobalt ferrite samples showed ferromagnetic behavior at room temperature, which is illustrated by the obtained hysteresis curves (Figure 18a,b). The main magnetic characteristics, such as coercive field and maximum magnetization, were determined from the hysteresis curves. The maximum magnetization value for the sample of pure cobalt ferrite CoFe_2_O_4_ is 61 emu/g (48 nm crystallites size), close to 67.7 emu/g (crystallites size about 88 nm) for CoFe_2_O_4_ obtained by wet-chemical synthesis [26].

The composite sample containing SiO_2_ and TiO_2_ has a very small maximum magnetization, around 3 emu/g, as the weight magnetic fraction in this sample is just around 1.2% (Table 2). The coercivity of the cobalt ferrite samples is relatively high, around 800 Oe, slightly higher for the sample containing SiO_2_ and TiO_2_, which could probably be explained by the different anisotropy due to the presence of the great amount of non-magnetic material in this sample. The magnetization of CoFe_2_O_4_/SiO_2_/TiO_2_ can hardly be compared with similar composites because of the difference in the applied magnetic field of 6 kOe applied by us, but 15 kOe in [26], and 20 kOe in [25]. Still, the maximum magnetization is related to the applied field of 3 emu/g for 6 kOe and 6.5 emu/g for 20 kOe [25]. On the other hand, zinc ferrite samples show superparamagnetic behavior at room temperature, with zero coercivity and a well-defined S-shaped M–H curve (Figure 18c,d). The superparamagnetic behavior of the zinc ferrite samples could be explained by the very small nanoparticles size, around 19 nm, as obtained from Rietveld’s method (See Table 2). The maximum magnetization for the pure zinc ferrite sample is 11 emu/g, and for the sample containing SiO_2_ and TiO_2_, it is 8 emu/g.

**Figure 19:**
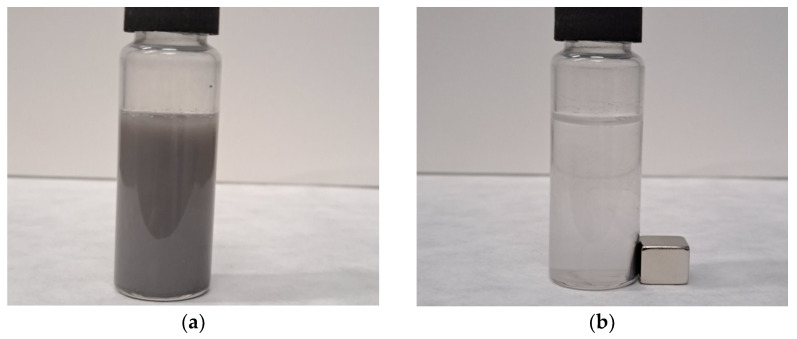
(**a**) Suspension containing the CoFe_2_O_4_/SiO_2_/TiO_2_(HT) catalyst; (**b**) solution and catalyst separated by an NdFeB magnet.

Using an NdFeB magnet with a size of 9 × 9 × 9 mm and power of 42.1 N, the ferromagnetic behavior of the cobalt ferrite samples at room temperature was confirmed. The successful separation of the suspension, containing the catalyst CoFe_2_O_4_/SiO_2_/TiO_2_(HT), prepared for the photocatalytic activity test for MG degradation, is presented in Figure 19.

## 3. Discussion

Nanocomposites of the “core–shell” type, e.g., CoFe_2_O_4_/SiO_2_, CoFe_2_O_4_/TiO_2_, CoFe_2_O_4_/SiO_2_/TiO_2_, and ZnFe_2_O_4_/SiO_2_/TiO_2_, were obtained using two different procedures for the synthesis of the MFe_2_O_4_ core: the sol-gel (SG), and hydrothermal (HT) methods.

(i) Photocatalytic activity of the CoFe_2_O_4_/TiO_2_ (SG) and CoFe_2_O_4_/SiO_2_/TiO_2_ (SG) composites. The comparison confirms the role of SiO_2_ as an insulator. The rate constant and the degradation of MG in the process are increasing, i.e., values of 2.9 × 10^−3^ min^−1^ (degradation 41%) for CoFe_2_O_4_/TiO_2_ (SG) and (SG) 5.8 × 10^−3^ min^−1^ (degradation 48%) for CoFe_2_O_4_/SiO_2_/TiO_2_ are obtained. A similar system to the kind used for CoFe_2_O_4_/SiO_2_/TiO_2_ can be experimented with for a different organic dye degradation, Methylene Blue [24], but due to the lack of any rate constant or percentage of degradation, any comparison with our results cannot be made.
(ii) Photocatalytic activity of the CoFe_2_O_4_/SiO_2_/TiO_2_ (SG) and CoFe_2_O_4_/SiO_2_/TiO_2_ (HT) composites. Above and beyond the different ferrite “core” MFe_2_O_4_ (M = Zn, Co), by XRD, some changes in the phase composition of the nanocomposites, mainly affecting the “shell” TiO_2_, were detected. Comparing the polymorphs of TiO_2_ in CoFe_2_O_4_/SiO_2_/TiO_2_(SG) and CoFe_2_O_4_/SiO_2_/TiO_2_(HT), it can be seen that the former contains only anatase, but the latter contains both anatase and rutile. The first one, CoFe_2_O_4_/SiO_2_/TiO_2_(SG), is more active, with a rate constant for MG decomposition under UV light irradiation of 5.8 × 10^−3^ min^−1^ (degradation 48%), while the latter, CoFe_2_O_4_/SiO_2_/TiO_2_(HT), is less active under irradiation of 2.6 × 10^−3^ min^−1^ (degradation 39%). The reason for the different activity can be connected with the different polymorphs of TiO_2_, anatase or anatase/rutile. According to [52,53], anatase demonstrates higher photocatalytic activity in comparison to polymorphs rutile and brookite because of more surface defects and oxygen vacancies in anatase compared to rutile, leading to charge separation and better photocatalytic performance; however, according to [54,55] a combination of A/R displays better photocatalytic activity than each of the polymorphs. A well-known example is the anatase/rutile mixture of TiO_2_-Degussa P25 with 80% anatase and 20% rutile, being one of the most investigated photocatalysts with good activity under UV light [55]. In CoFe_2_O_4_/SiO_2_/TiO_2_(HT), the weight fraction (%) of anatase is 87.7 and of rutile is 11.1, apparently differing from that of TiO_2_-Degussa P25.
(iii) The ZnFe_2_O_4_/SiO_2_/TiO_2_(HT) and CoFe_2_O_4_/SiO_2_/TiO_2_(HT) composites. Similarly, ZnFe_2_O_4_/SiO_2_/TiO_2_(HT) contains anatase, while in CoFe_2_O_4_/SiO_2_/TiO_2_(HT), both anatase and rutile are detected, with the weight fraction mentioned (%), A/R 87.7/11.1, differing from that of TiO_2_-Degussa P25. The rate constant for MG decomposition is 9 × 10^−3^ min^−1^ (degradation 80%) for ZnFe_2_O_4_/SiO_2_/TiO_2_(HT), while for the process with CoFe_2_O_4_/SiO_2_/TiO_2_(HT), 2.6 × 10^−3^ min^−1^ (degradation 39%) was obtained.
(iv) Weight ratio of A/R. This happens to be crucial in order to overcome the photocatalytic activity of anatase [56,57]. It is found that an A/R mixture of 90/10 has a weak activity for the decomposition of Methylene Blue, while a mixture of 86/14 is more effective in the same process than commercial TiO_2_ Degussa P25 [58]. So, our results confirm that not only is the mixture of anatase/rutile essential, but the weight fraction of the polymorphs is also important. For example, the rate constant for the process of RhB degradation by CoFe_2_O_4_/SiO_2_/TiO_2_ (HT) is close to one obtained [59] for TiO_2_ containing A/R. However, such a comparison is not correct because of the missing exact value for the A/R ratio in [59]. In addition, differences in the catalyst dose, the pollutant concentration, and the irradiation time should be taken into account. All this is illustrated by the data included in Table S1 [60,61,62,63,64] and Table S2 [59,65].

Out of the three composites, CoFe_2_O_4_/SiO_2_/TiO_2_ (SG), CoFe_2_O_4_/SiO_2_/TiO_2_ (HT), and ZnFe_2_O_4_/SiO_2_/TiO_2_ (HT), the latter displays the highest photocatalytic activity for degradation of MG both under UV and simulated solar light, having a high content of anatase on the surface of the core/shell structure.

Despite the lower activity of CoFe_2_O_4_/SiO_2_/TiO_2_ (HT), it still possesses valuable ferromagnetic properties with magnetization of 3 emu/g, which increases its potential in practical applications.

## 4. Materials and Methods

### 4.1. Materials

The chemicаls Co(NO_3_)_2_·6H_2_O, Fe(NO_3_)_3_·9H_2_O (p.а., Sigma-Aldrich, Milwaukee, WI, USA), tetraethyl orthosilicate, TEOS (reagent grade, 98%, Sigma-Aldrich, Milwaukee, WI, USA), Ti(IV) tetrabutoxide, and Ti(OBu)_4_ (reagent grade, 97%, Sigma-Aldrich, Milwaukee, WI, USA) were used in this study as well as the dyes Malachite Green oxalate (MG), (Chroma GmbH, Hamburg, Germany) and Rhodamin B (RhB). An NdFeB magnet (MAGSY, s.r.o., Fryšták, Czech Republic) with a size of 9 × 9 × 9 mm and power 42.1 N was used.

### 4.2. Synthetic Procedures

#### 4.2.1. Synthesis of CoFe_2_O_4_ by the Sol-Gel Method

Co(NO_3_)_2_·6H_2_O and Fe(NO_3_)_3_·9H_2_O were dissolved in 96 wt% ethanol with a mole ratio n(Co^2+^):n(Fe^3+^) = 1:2 and stirred until complete homogenization for 30 min, then propylene oxide (PO) was added as a gelling agent (ratio PO/EtOH = 1: 4) and stirred for an additional 30 min for sol formation. The sol was kept at 200 °C for 24 h until a gel was formed. After that, the prepared sample of CoFe_2_O_4_ was annealed at 550 °C for 5 h.

#### 4.2.2. Synthesis of CoFe_2_O_4_ and ZnFe_2_O_4_ by Hydrothermal Method

The metal salts Co(NO_3_)_2_·6H_2_O and Fe(NO_3_)_3_·9H_2_O with mole ratio n(Co^2+^):n(Fe^3+^) = 1:2 were dissolved in 50 mL of ethylene glycol, EG. The solution was stirred for 30 min by magnetic stirring, followed by 30 min sonication in an ultrasonic bath. After adding NaOH and stirring for 30 min, the metal ions were precipitated. The mixture was transferred to a 75 mL PTFE autoclave and kept at 180 °C for 24 h. The samples obtained wеre annealed at 550 °C for 5 h.

#### 4.2.3. Coating of MFe_2_O_4_ (M = Zn(II), Co(II)) with SiO_2_ and TiO_2_

Coating with layers of SiO_2_ and TiO_2_ was performed in ethyl alcohol media, as initial substances tetraethyl orthosilicate, TEOS, Ti(IV)tetrabutoxide, and Ti(OBu)_4_ were used, respectively.

In order to form a layer of SiO_2_, the quantity of CoFe_2_O_4_ and TEOS was calculated to obtain a mole ratio of n(CoFe_2_O_4_) /nSiO_2_ = 0.01. The choice of that ratio was a result of preliminary experiments not discussed here, and it was considered optimal for successful SiO_2_ layer formation on the basis of TEM micrographs of the samples produced (Figure 2). Briefly, to a suspension of CoFe_2_O_4_ in pure ethanol, TEOS was added (volume ratio TEOS:EtOH = 0.025), and it was stirred until homogenization was achieved. In addition, the NH_3_ water solution was added drop-wise up to pH 12, so basic hydrolysis was applied. Stirring continued at 20 °C for 24 h. After that, the samples were washed two-fold in a small amount of ethanol and kept at 60 °C for 48 h for drying. An analogous sample with ZnFe_2_O_4_ was not included among the samples characterized.

After SiO_2_ layer deposition, a layer of TiO_2_ was deposited on CoFe_2_O_4_/SiO_2_. For this purpose, a pure ethanol sample of CoFe_2_O_4_/SiO_2_ was suspended, Ti(IV) tetrabutoxide was dissolved (volume ratio Ti(OBu)_4_:EtOH = 0.025), and after homogenization, NH_3_ water solution was added drop-wise. The procedure included stirring for 24 h at 20 °C, washing with pure EtOH, and drying for 24 h at 60 °C. After calcination for 5 h at 550 °С, the sample considered to be CoFe_2_O_4_/SiO_2_/TiO_2_ was further characterized and tested. An identical procedure for ZnFe_2_O_4_/SiO_2_/TiO_2_ and CoFe_2_O_4_/TiO_2_ formation was applied.

The “core/shell” of the MFe_2_O_4_/SiO_2_/TiO_2_ type contained MFe_2_O_4_ (M = Zn or Co) prepared by two different synthetic methods, the sol-gel (SG) and hydrothermal (HT) method. For the purpose of distinguishing, they are named in the text, including the initials of the synthetic methods, SG or HT.

### 4.3. Methods for Characterization

X-ray powder diffraction (XRD) patterns for phase identification were recorded at an angle interval of 10–80° (2θ) on a Philips PW 1050 diffractometer equipped with a CuKα tube and scintillation detector. Data for cell refinements were collected in θ–2θ, step-scan mode at an angle interval from 10 to 90° (2θ), at steps of 0.02° (2θ), and with a counting time of 1 s/step. Phase identification was carried out using Match! 3 software [66] coupled with the Crystallography Open Database [67]. The unit cell parameters, crystallite size, and phase weight fraction were extracted by the Rietveld method using the MAUD program [68].

A transmitting electron microscope with energy dispersive X-ray spectroscopy EDS was used with a high resolution (HRTEM), JEM 2100 (JEOL), 200 kV, and up to 1,500,000 times magnification was applied to follow the morphology of the samples.

X-ray photoelectron spectroscopy (XPS) was performed using a VG Escalab MKII electron spectrometer with achromatic AlKα radiation and energy of 1486.6 eV. The binding energies correction was evaluated by utilizing the C 1s line as a reference with an energy of 285.0 eV. The photoelectron lines of constituent elements on the surface were recorded and corrected by subtracting a Shirley-type background. The deconvolution of spectra was carried out with XPSPEAK41 software.

To carry out UV-Vis absorption spectroscopy, an Evolution 300 UV-Vis spectrometer (Thermo Fisher Scientific Inc., Waltham, MA, USA ) was used to measure the absorption of the samples within a range of 200–900 nm.

Band gap energies were calculated from the UV-Vis absorption spectra ranging from 200 to 1000 nm. The UV–Vis data were analyzed to determine the relation between the optical band gap, absorption coefficient, and energy (*hν*) of the incident photon for near-edge optical absorption in semiconductors. The band gap energy was calculated from the measured curves by fits according to Tauc’s equation *αhν = A(hν − Eg)^n/2^*, where *A* is a constant independent of *hν*, *Eg* is the semiconductor band gap, and *n* depends on the type of transition [69]. The value used for n was 1, reflecting a direct transition. The well-known approach for semiconductor band gap energy determination from the intersection of linear fits of (*αhν*)^1/n^ versus *hv* on the x-axis was used, where n can be 1/2 and 2 for direct and indirect band gaps, respectively.

Photoluminescent spectroscopy was applied to record the photoluminescence spectra of the obtained catalysts at 315 nm using a FluoroLog3-22, Horiba JobinYvon spectrophotometer.

Magnetic properties measurements. Magnetization curves of the powdered samples were measured at room temperature using a vibrating sample magnetometer (VSM) in a magnetic field up to 6 kOe. The powders were pressed into cylindrical quartz containers so that the particles were fixed during the measurements.

### 4.4. Photocatalytic Activity

The activity of the samples as photocatalysts for the degradation of Malachite Green and Rhodamine B in model water solutions under UV light and simulated solar irradiation was tested. The photocatalytic tests were performed following the procedure described in [3,17]. Briefly, in a 500 mL Lenz Laborglas glass batch slurry reactor (model LF100) (1 g catalyst/L), a 200 mL 10^−5^ M aqueous solution of Malachite Green/(Rhodamine B) was used as a model pollutant. After a 30 min ‘‘dark’’ period (in order to establish the equilibrium of the sorption process), the system was UV-illuminated by a lamp (Sylvania 18 W BLB T8, emission in the 345–400 nm region with a maximum at 365 nm), situated at 9.5 cm distance above the slurry (illumination intensity 0.5 W/m) under continuous magnetically stirring (400 min^−1^) and bubbling with air (45 L/h). The role of the bubbling with air was to ensure the excess of oxygen was that which wasstoichiometrically needed. As a light source, a sunlight simulator 300 W lamp (Osram, Munich, Germany, Ultra Vitalux) was used. This is a UV/Vis lamp, with radiated power in the region of 315–400 nm (UVA) 13.6 W and in the region of 280–315 nm (UVB) 3.0 W. Spectral radiation distribution shows a high portion of the UV radiation [46]. The lamp was situated 15 cm above the suspension. A Pt/Rh thermocouple was inserted into the reaction mixture and connected to the ArgoLab CB-5 thermostat for better temperature control. The temperature was kept at 25 °C ± 0.1 during the whole procedure, including during the sorption–desorption (“dark”) period. The initial pH of the solution was between 5.8 and 5.9. Periodically, a 5-mL aliquot was taken from the solution and filtered through a 0.20-µm Minisart filter. The dye concentration was determined spectrophotometrically by the band at 622 nm (for MG) or 550 nm (for RhB). The data obtained were plotted in coordinates (C/C_0_)/t and −ln(C/C_0_)/t (where C_0_ is the concentration after the ‘‘dark’’ period, and C is the concentration after t min irradiation). The apparent rate constants of the degradation process were determined assuming first-order kinetics. The sorption capacity was calculated as the ratio of (C_00_–C_0_)/C_00_, where C_00_ is the starting solution concentration (before the ‘‘dark’’ period). The dye degradation in moment t was determined by the following formula: degradation,% = (Аo − Аt)/Аo × 100, where Аo is the initial absorption of the dye solution at moment t = 0 min, and Аt is the absorption at moment t min.

### 4.5. Photocatalytic Activity in Real Water Matrix

As Sofia University (located in Sofia city) does not have easy access to large water bodies, artificial seawater was used. The artificial seawater was prepared following the procedure proposed by [70]. It contained NaCl (25 g/L), MgCl_2_ (11 g/L), Na_2_SO_4_ (4 g/L), and CaCl_2_ (1.6 g/L) in ultrapure water.

## 5. Conclusions

Nanocomposites of the type МFe_2_O_4_/SiO_2_/TiO_2_ (M = Zn(II), Co(II)), with superparamagnetic and ferromagnetic properties, were synthesized, and their “core/shell” structure and morphology were proven. The structural and microstructural characteristics of the samples (crystallite size, microstrains, and weight fraction of the phases) were obtained by Rietveld analysis of the XRD data of the composites. By the combination of both experimental techniques, TEM/EDS and XPS, the phase composition and the structure of the “core/shell” were evidenced. Among the methods used for characterization, the XPS technique, known to be surface-sensitive, showed limitations for the detection of the elements in the core of the “core/shell” (Fe, Zn/Co) due to the thickness of the layers on the surface of the core.

The highest degradation efficiency of 98% and the highest rate constants were achieved for the degradation of MG and RhB under simulated solar light due to the high portion of UV radiation in the solar light simulator. The investigated influence of the water matrix on the photocatalytic activity showed that artificial seawater inhibited the role of both the UV and simulated solar light. As a result, decreased efficiency of the degradation and lower values of the rate constants of the photocatalytic process were obtained.

The detected decrease in the composite’s maximum magnetization in comparison with pure CoFe_2_O_4_ is due to the contribution of non-magnetic shells. The SiO_2_/TiO_2_ layers on CoFe_2_O_4_ are magnetically inactive, disturbing the magnetization of the composite.

The charge separation, important for the photocatalytic activity, was registered by photoluminescence measurements. Despite the improved charge separation of the composites in comparison with TiO_2_, their photocatalytic activity was lower than that of TiO_2_ P25. This is quite likely due to the anatase/rutile ratio for MFe_2_O_4_/SiO_2_/TiO_2_, differing from that of TiO_2_-Degussa P25. Despite the lower photocatalytic activity of the CoFe_2_O_4_/SiO_2_/TiO_2_ (HT) nanocomposite, it possessed a magnetic saturation of 3 emu/g. Samples of this kind can be applied to separate the photocatalyst from the purified water. Based on the available literature data, we may well affirm that the work presented here is the first study on the photocatalytic degradation of MG and RhB by MFe_2_O_4_/SiO_2_/TiO_2_ (M = Zn, Co) composites.

## Figures and Tables

**Table 1:** Unit cell parameters and crystallite size of CoFe_2_O_4_, produced via the sol-gel method, and the nanocomposites on its base.

Sample	Unit Cell Parameters, Å		Crystallite Size, nm
CoFe_2_O_4_ (SG)	a = 8.3612(2)		48.1 (2)
CoFe_2_O_4_/TiO_2_ (SG)	Anatase	a = 3.7833 (1) c = 9.4391 (3)	25.3 (3)
	Rutile	a = 4.5815 (2) c = 2.9531 (4)	20.1 (2)
	CoFe_2_O_4_	a = 8.3652 (10)	56.6 (7)
CoFe_2_O_4_/SiO_2_/TiO_2_ (SG)	Anatase	a = 3.7893 (1) c = 9.4364 (6)	16.8 (6)
	CoFe_2_O_4_	a = 8.3631 (9)	53.1 (4)

**Table 2:** Lattice constants and crystallite size of the nanocomposites produced via the HT method.

	ZnFe_2_O_4_/SiO_2_/TiO_2_ (HT)		CoFe_2_O_4_/SiO_2_/TiO_2_ (HT)		
	ZnFe_2_O_4_	Anatase	CoFe_2_O_4_	Rutile	Anatase
Unit cell parameters, Å	a = b = c = 8.4463 (9)	A = b = 3.7899 (2) c = 9.5230 (7)	a = b = c = 8.3874 (15)	a = b = 4.5953 (6) c = 2.9608 (7)	a = b = 3.7860 (2) c = 9.5185 (7)
Crystallite size, nm	19.3 (5)	41.2 (1)	34.2 (3)	42.8 (9)	36.8 (3)
Microstrains, ×10^−3^ a.u.	2.6 (3)	2.0 (2)	2.6 (5)	0.6 (1)	1.6 (3)
Weight fraction,%	19.5 ± 2.1	80.5 ± 2.1	1.2 ± 0.3	11.1 ± 1.1	87.7 ± 5.1
R_wp_,%	5.64		7.37		
χ^2^	1.10		1.18		

**Table 3:** Photocatalytic activity of ZnFe_2_O_4_/SiO_2_/TiO_2_ (HT) in ultrapure and artificial water matrices under UV light and simulated solar irradiation.

Ultrapure H_2_O								Artificial Sea H_2_O							
UV Light				Simulated Solar Light				UV Light				Simulated Solar Light			
Rate Constant, 10^−3^ min^−1^		Degradation, %		Rate Constant, 10^−3^ min^−1^		Degradation, %		Rate Constant, 10^−3^ min^−1^		Degradation, %		Rate Constant, 10^−3^ min^−1^		Degradation, %	
MG	RhB	MG	RhB	MG	RhB	MG	RhB	MG	RhB	MG	RhB	MG	RhB	MG	RhB
9.0	-	80	53	77	65	98	98	5	3.6	59.5	42	5.7	4.0	93	90

## Data Availability

Data are contained within the article.

## Supplementary Materials

The following supporting information can be downloaded at https://www.mdpi.com/article/10.3390/ijms25126755/s1.

## References

[1] Linsebigler A.L., Lu G., Yates J.T. (1995). Photocatalysis on TiO_2_ surfaces: Principles, mechanisms and selected results. Chem. Rev..

[2] Xu D., Ma H. (2021). Degradation of rhodamine B in water by ultrasound-assisted TiO_2_ photocatalysis. J. Clean. Prod..

[3] Kralchevska R., Milanova M., Hristov D., Pintar A., Todorovsky D. (2012). Synthesis, characterization and photocatalytic activity of neodymium, nitrogen and neodymium-nitrogen doped TiO_2_. Mater. Res. Bull..

[4] Zhou X., Shi T.J., Zhou H.O. (2012). Hydrothermal preparation of ZnO-reduced graphene oxide hybrid with high performance in photocatalytic degradation. Appl. Surf. Sci..

[5] Rahman Q.I., Ahmad M., Misra S.K., Lohani M. (2013). Effective photocatalytic degradation of Rhodamine B dye by ZnO nanoparticles. Mater. Lett..

[6] Lee K.M., Lai C.W., Ngai K.S., Juan J.C. (2016). Recent developments of zinc oxide based photocatalyst in water treatment technology: A review. Water Res..

[7] Ong C.B., Ng L.Y., Mohammad A.W. (2018). A review of ZnO nanoparticles as solar photocatalysts: Synthesis, mechanisms and applications. Renew. Sustain. Energy Rev..

[8] Vasei H.V., Masoudpanah S.M., Pouya V.K. (2021). Photocatalytic activity of solution combustion synthesized ZnO powders by using a mixture of DTAB and citric acid fuels. J. Phys. Chem. Solids.

[9] Qian L., Zhu J., Du W., Qian X. (2009). Solvothermal synthesis, electrochemical and photocatalytic properties of monodispersed CeO_2_ nanocubes. Mater. Chem. Phys..

[10] Bellardita M., Fiorenza R., Palmisano L., Scirè S. (2020). Photocatalytic and photothermocatalytic applications of cerium oxide-based materials. Cerium Oxide (CeO₂): Synthesis, Properties and Applications.

[11] Wan X., Yuan M., Tie S.-I., Lan S. (2013). Effects of catalyst characters on the photocatalytic activity and process of NiO nanoparticles in the degradation of methylene blue. Appl. Surf. Sci..

[12] Chauhan R., Kumarb A., Chaudhary R.P. (2013). Visible-light photocatalytic degradation of methylene blue with Fe doped CdS nanoparticles. Appl. Surf. Sci..

[13] Zhang H.L., Wei B., Zhu L., Yua J.H., Sun W.J., Xu L.L. (2013). Cation exchange synthesis of ZnS-Ag_2_S microspheric composites with enhanced photocatalytic activity. Appl. Surf. Sci..

[14] Casbeer E., Sharma V.K., Li X.-Z. (2012). Synthesis and photocatalytic activity of ferrites under visible light: A review. Sep. Purif. Technol..

[15] Ch K., Dhar S.S. (2020). Rapid catalytic degradation of malachite green by MgFe_2_O_4_ nanoparticles in presence of H_2_O_2_. J. Alloys Compd..

[16] Krishnamoorthi A.M.C., Babu K.C., Pavithra N.C. (2019). Dielectric, magnetic hyperthermia, and photocatalytic properties of ZnFe_2_O_4_ nanoparticles synthesized by solvothermal reflux method. Appl. Phys. A.

[17] Tsvetkov M., Zaharieva J., Milanovа M. (2020). Ferrites, modified with silver nanoparticles, for photocatalytic degradation of malachite green in aqueous solutions. Catal. Today.

[18] Manohar A., Chintagumpala K., Hyeon K.K. (2021). Mixed Zn–Ni spinel ferrites: Structure, magnetic hyperthermia and photocatalytic properties. Ceram. Intern..

[19] Lassoued A., Li J.F. (2020). Magnetic and photocatalytic properties of Ni–Co ferrites. Solid State Sci..

[20] Dojcinovic M.P., Vasiljevic Z.Z., Pavlovic V.P., Barisic D., Pajic D., Tadic N.B., Nikolic M.V. (2021). Mixed Mg–Co spinel ferrites: Structure, morphology, magnetic and photocatalytic properties. J. Alloys Compd..

[21] Kurian M., Thankachan S. (2021). Structural diversity and applications of spinel ferrite core—Shell nanostructures—A review. Open Ceram..

[22] Klekotka U., Piotrowska B., Satuła D., Kalska-Szostko B. (2018). Modified ferrite core-shell nanoparticles magneto-structural characterization. Appl. Surf. Sci..

[23] Masala O., Hoffman D., Sundaram N., Page K., Proffen T., Lawes G., Seshadri R. (2006). Preparation of magnetic spinel ferrite core/shell nanoparticles: Soft ferrites on hard ferrites and vice versa. Solid State Sci..

[24] Greene D., Serrano-Garcia R., Govan J., Gun’ko Y.K. (2014). Synthesis Characterization and Photocatalytic Studies of Cobalt Ferrite-Silica-Titania Nanocomposites. Nanomaterials.

[25] Hamad H., Abd El-Latif M., Kashyout A.E.-H., Sadik W., Feteha M. (2015). Synthesis and characterization of core–shell–shell magnetic (CoFe_2_O_4_–SiO_2_–TiO_2_) nanocomposites and TiO_2_ nanoparticles for the evaluation of photocatalytic activity under UV and visible irradiation. N. J. Chem..

[26] Harraz F.A., Mohamed R.M., Rashad M.M., Wang Y.C., Sigmund W. (2014). Magnetic nanocomposite based on titania–silica/cobalt ferrite for photocatalytic degradation of methylene blue dye. Ceram. Int..

[27] Ren X., Chen X., Ding H., Yang H. (2015). Luminescent and magnetic properties of CoFe_2_O_4_@SiO_2_@Y_2_O_3_:Tb^3+^ nanocomposites with the core–shell. J. Alloys Compd..

[28] Dee G., Shayoub H., McNeill H., Sánchez Lozano I., Rafferty A., Gun’ko Y.K. (2023). MnFe_2_O_4_@SiO_2_@CeO_2_ core–shell nanostructures for applications in water remediation. RSC Adv..

[29] Rao V.N., Reddy N.L., Preethi V., Karthik M., Yu Y.-T., Yang J.M., Kumari M.M., Shankar M.V. (2023). A critical review on core/shell-based nanostructured photocatalysts for improved hydrogen generation. Int. J. Hydrogen Energy.

[30] Lee C.-T., Lee H.-Y., Chen H.-W. (2003). GaN MOS device using SiO_2_ insulator grown by photoelectrochemical oxidation method. IEEE Electron Device Lett..

[31] Peng T., Zhao D., Dai K., Shi W., Hirao K. (2005). Synthesis of titanium dioxide nanoparticles with mesoporous anatase wall and high photocatalytic activity. J. Phys. Chem. B.

[32] Gong X.-Q., Selloni A. (2005). Reactivity of anatase TiO_2_ nanoparticles: The role of the minority (001) surface. J. Phys. Chem. B.

[33] Zhang W.F., He Y.L., Zhang M.S., Yin Z., Chen Q. (2000). Raman scattering study on anatase TiO_2_ nanocrystals. J. Phys. D.

[34] Yadav A.A., Kang S.-W., Hunge Y.M. (2021). Photocatalytic degradation of Rhodamine B using graphitic carbon nitride photocatalyst. J. Mater. Sci. Mater. Electron..

[35] Sutili F.J., Gressler L.T. (2021). Antimicrobial agents. Aquaculture Pharmacology.

[36] Hanaor D.A.H., Sorrell C.C. (2011). Review of the anatase to rutile phase transformation. J. Mater. Sci..

[37] Nolan N.T., Synnott D.W., Seery M.K., Hinder S.J., Van Wassenhoven A., Pillai S.C. (2012). Effect of N-doping on the photocatalytic activity of sol–gel TiO_2_. J. Hazard. Mater..

[38] Kralchevska R., Milanova M., Kovacheva P., Kolev J., Avdeev G., Todorovsky D. (2011). In-fluence of ThO_2_ on the photocatalytic activity of TiO_2_. Cent. Eur. J. Chem..

[39] Engelhard M.H., Droubay T.C., Du Y. (2017). X-ray Photoelectron Spectroscopy Applications. Encyclopedia of Spectroscopy and Spectrometry.

[40] Wing M., Gong B., Yao X., Wang Y., Lamb R.N. (2006). Preparation and microstructure properties of Al-doped TiO_2_-SiO_2_ gel-glass film. Thin Solid Film..

[41] Manole A.V., Dobromir M., Apetrei R., Nica V., Lucaet D. (2014). Surface characterization of sputtered N:TiO_2_ thin films within a wide range of dopant concentration. Ceram. Int..

[42] Guo N., Liang Y., Lan S., Liu L. (2014). Uniform TiO_2_–SiO_2_ hollow nanospheres: Synthesis, characterization and enhanced adsorption–photodegradation of azo dyes and phenol. Appl. Surf. Sci..

[43] Peng T., Ray S., Veeravalli S.S., Lalman J.A., Arefi-Khonsari F. (2018). The role of hydrothermal conditions in determining 1D TiO_2_ nanomaterials bandgap energies and crystal phases. Mater. Res. Bull..

[44] Ray S.K., Dhakal D., Lee S.W. (2018). Insight into Malachite Green Degradation, Mechanism and Pathways by Morphology-Tuned α-NiMoO_4_ Photocatalyst. Photochem. Photobiol..

[45] Weber M., Schulmeister K. (2003). Hazard Assessment of Lamps following the CIE Lamp Safety Standard. https://laser-led-lamp-safety.seibersdorf-laboratories.at/fileadmin/uploads/intranet/2003%20cie%20hazard%20assessment%20of%20lamps_%20weber.pdf.

[46] Stefan M. (2016). More Than Just Light. Solutions in Ultraviolet Light. 100 Years of Innovation Osram. https://docs.rs-online.com/45d7/0900766b81506d14.pdf.

[47] Hu L., Wang P., Zhang G., Liu G., Li Y., Shen T., Crittenden J.C. (2020). Enhanced persulfate oxidation of organic pollutants and removal of total organic carbons using natural magnetite and microwave irradiation. Chem. Eng. J..

[48] Schneider J.T., Firak D.S., Ribeiro R.R., Peralta-Zamora P. (2020). Use of scavenger agents in heterogeneous photocatalysis: Truths, half-truths, and misinterpretations. Phys. Chem. Chem. Phys..

[49] Denisov N., Yoo J.E., Schmuki P. (2019). Effect of different hole scavengers on the photoelectrochemical properties and photocatalytic hydrogen evolution performance of pristine and Pt-decorated TiO_2_ nanotubes. Electrochim. Acta.

[50] Slapničar Š., Žerjav G., Zavašnik J., Finšgar M., Pintar A. (2023). Synthesis and characterization of plasmonic Au/TiO_2_ nanorod sol-ids for heterogeneous photocatalysis. J. Environ. Chem. Eng..

[51] Serpone N., Lawless D., Khairutdinov R. (1995). Size effects on the photophysical properties of colloidal anatase TiO_2_ particles: Size quantization or direct transition in this indirect semiconductor?. J. Phys. Chem..

[52] Zhang J., Zhou P., Liu J., Yu J. (2014). New understanding of the difference of photocatalytic activity among anatase, rutile and brookite TiO_2_. Phys. Chem. Chem. Phys..

[53] Golubović A., Scepanovic M.J., Kremenovic A. (2009). Raman study of the variation in anatase structure of TiO_2_ nanopowders due to the changes of sol-gel synthesis conditions. J. Sol-Gel Sci. Technol..

[54] Hurum D.C., Agrios A.G., Gray K.A., Rajh T., Thurnauer M.C. (2003). Explaining the Enhanced Photocatalytic Activity of Degussa P25 Mixed-Phase TiO_2_ Using EPR. J. Phys. Chem. B.

[55] Yu J., Yu H., Cheng B., Zhou M., Zhao X. (2006). Enhanced photocatalytic activity of TiO_2_ powder (P25) by hydrothermal treatment. J. Mol. Catal. A Chem..

[56] Arbuj S.S., Hawaldar R.R., Mulik U.P., Waniet B.N., Amalnerkar D.P., Waghmode S.B. (2010). Preparation, characterization and photocatalytic activity of TiO_2_ towards methylene blue degradation. Mater. Sci. Eng. B.

[57] Yuangpho N., Lee S.T.T., Treerujiraphapong T., Khanitchaidecha W., Nakaruk A. (2015). Enhanced photocatalytic performance of TiO_2_ particles via effect of anatase–rutile ratio. Phys. E Low-Dimens. Syst. Nanostructures.

[58] Nolan N.T., Seery M.K., Pillai S.C. (2009). Spectroscopic investigation of the anatase-to- rutile transformation of sol–gel-synthesized TiO_2_ photocatalysts. J. Phys. Chem. C.

[59] Mahlambi M.M., Mishra A.K., Mishra S.B., Krause R.W., Mamba B.B., Raichur A.M. (2012). Comparison of Rhodamine B degradation under UV irradiation by two phases of titania nano-photocatalyst. J. Therm. Anal. Calorim..

[60] Bachvarova-Nedelcheva A., Iordanova R., Georgieva N., Nemska V., Foteva T., Stoyanova A. (2024). Influence of Nb_2_O_5_ and B_2_O_3_ on the photocatalytic and antibacterial activity of sol-gel derived TiO_2_ nanopowders. J. Chem. Techn. Metall..

[61] Bachvarova-Nedelcheva A., Iordanova R., Naydenov A., Stoyanova A., Georgieva N., Nemska V., Foteva T. (2023). Sol-Gel Obtaining of TiO_2_/TeO_2_ Nanopowders with Biocidal and Environmental Applications. Catalysts.

[62] Tarigh G.D., Shemirani F., Mazhari N.S. (2015). Fabrication of a reusable magnetic multi-walled carbon nanotube–TiO_2_ nanocomposite by electrostatic adsorption: Enhanced photodegradation of malachite green. RSC Adv..

[63] Atalı Ş.G., Akbal F., Gürbüz M., Turan B.C. (2023). Granular titanium dioxide and silicon-doped titanium dioxide as reusable photocatalysts for dye removal. Int. J. Appl. Ceram. Technol..

[64] Ivanova D., Mladenova E., Kaneva N. (2024). Photo-fixation of Ag onto TiO_2_ powder films for improved photocatalytic activity and atomic-absorption determination of residual noble metal in dyes solution. C. R. Acad. Bulg. Sci..

[65] Aarthi T., Madras G. (2007). Photocatalytic Degradation of Rhodamine Dyes with Nano-TiO_2_. Ind. Eng. Chem. Res..

[66] Putz H., Brandenburg K. *Match!—Phase Analysis Using Powder Diffraction, Version 3.x, Crystal Impact*; GbR, Kreuzherrenstr: Bonn, Germany; Volume 102, p. 53227. https://www.crystalimpact.de/match.

[67] Grazulis S., Chateigner D., Downs R.T., Yokochi A.T., Quiros M., Lutterotti L., Manakova E., Butkus J., Moeck P., Le Bail A. (2009). Crystallography Open Database—An open-access collection of crystal structures. J. Appl. Crystallogr..

[68] Lutterotti L. (2000). Maud: A Rietveld analysis program designed for the internet and experiment integration. Acta Crystallogr. Sect. A Found. Crystallogr..

[69] Tauc J., Grigorovici R., Vancu A. (1966). Optical Properties and Electronic Structure of Amorphous Germanium. Phys. Status Solidi.

[70] Hu J., Sun C., Wu L., Zhao G., Liu H., Jiao F. (2023). Halogen doped g-C3N4/ZnAl-LDH hybrid as a Z-scheme photocatalyst for efficient degradation for tetracycline in seawater. Sep. Purif. Technol..

