# Supplementary material for: “Core/Shell” Nanocomposites as Photocatalysts for the Degradation of the Water Pollutants Malachite Green and Rhodamine B
Source: Int J Mol Sci. 2024 Jun 19;25(12):6755. doi: 10.3390/ijms25126755 (PMC11203973; doi:10.3390/ijms25126755)
Supplement: Supplementary file 1 [file ijms-25-06755-s001.zip › ijms-2999712-supplementary.pdf]

"CORE/SHELL" NANOCOMPOSITES BASED ON FERRITES AS PHOTOCATALYSTS FOR DEGRADATION OF THE WATER POLLUTANTS MALACHITE GREEN AND RHODAMINE B

J. Zaharieva[1], M. Tsvetkov[1*], M. Georgieva[2], D. Tsankov[2], M. Milanova[1*]

[1]Faculty of Chemistry and Pharmacy, Sofia University "St. Kliment Ohridski", 1, J. Bourchier, 1164 Sofia, Bulgaria; nhjz@chem.uni-sofia.bg
[2]Faculty of Physics, Sofia University "St. Kliment Ohridski", 5a, J. Bourchier, 1164 Sofia, Bulgaria; mgeorgieva@phys.uni-sofia.bg
*Correspondence: MM nhmm@chem.uni-sofia.bg ; MTs nhmt@chem.uni-sofia.bg

SUPPLEMENTARY INFORMATION

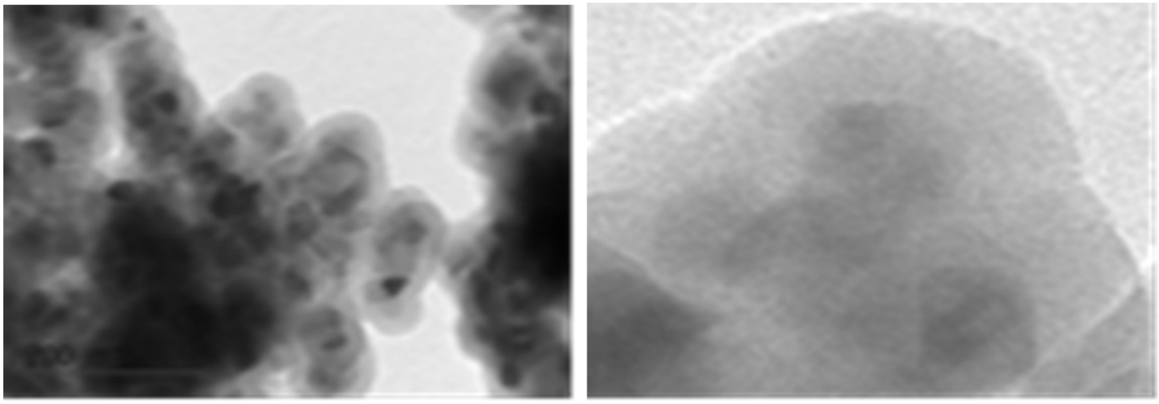

Figure S1. TEM-micrographs of the composite of the type core/shell $CoFe_2O_4/SiO_2$

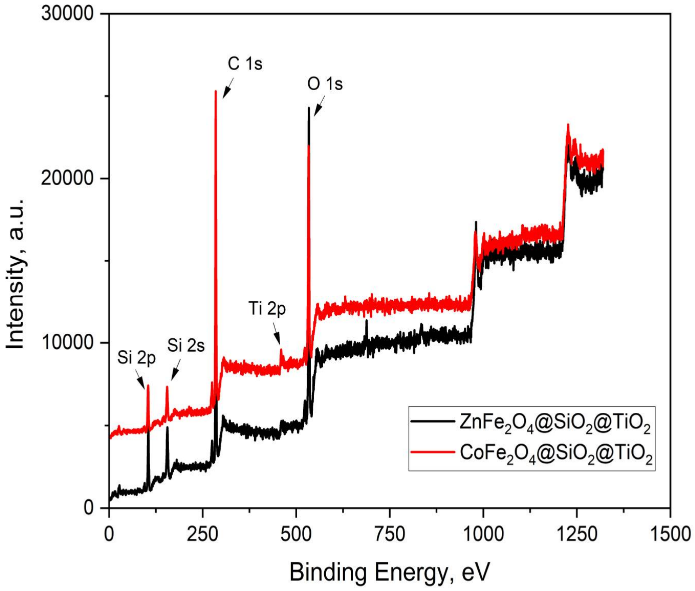

Figure S2. Low-energy-resolution wide-scan XPS spectra of "core-shell"composites $ZnFe_2O_4/SiO_2/TiO_2$(HT) and $CoFe_2O_4/SiO_2/TiO_2$(HT) (according the legend).

Table S1. Degradation of MG in model solution by TiO_2 and modified TiO_2 under UV irradiation

	Photo catalyst*	Catalyst dose, g L^{-1}	MG, mg L^{-1}	Irradiation, min	Degra dation, %	Rate constant, × 10^{-3} min^{-1}	Ref.
1	TiO_2-A	0.66	5	60	100	6.0	[60]
2	TiO_2-A,R	0.66	5	120	60	6.5	[61]
3	TiO_2-A,R/TeO_2	0.66	5	120	60	7.2	[61]
4	TiO_2-A/B_2O_3	0.66	5	90	75	17.2	[60]
5	CNT/TiO_2	0.2	50	240	90	-	[62]
6	SiO_2/TiO_2	50	10	60	91	-	[63]
7	TiO_2 -A/Ag	films	5	240	96	5.2	[64]
8	$ZnFe_2O_4$/SiO_2/TiO_2-A	1	5	180	80	9.0	This work
9	$CoFe_2O_4$/SiO_2/TiO_2-A,R	1	5	180	39	2.6	This work

*- A – anatase, R – rutile, CNT – carbon nanotubes.

Table S2. Degradation of RhB in model solution by TiO_2 and modified TiO_2 under UV irradiation

	Photo catalyst*	Catalyst dose, g L^{-1}	RhB, mg L^{-1}	Irradiation, min	Degra dation, %	Rate constant, × 10^{-3} min^{-1}	Ref.
1	TiO_2-A	0.1	10	30	59	17.0	[59]
2	TiO2-A,R	0.1	10	30	1.4	2.4	[59]
3	TiO_2-A,R	1	10			14.6	[65]
4	CS-TiO_2	1	10			28.7	[65]
5	$ZnFe_2O_4$/SiO_2/TiO_2-A	1	5	180	53		This work
6	$CoFe_2O_4$/SiO_2/TiO_2-A,R	1	5	180		1.11	This work

*- A – anatase, R – rutile.
